# BtubA-BtubB Heterodimer Is an Essential Intermediate in Protofilament Assembly

**DOI:** 10.1371/journal.pone.0007253

**Published:** 2009-09-29

**Authors:** Christopher A. Sontag, Harvey Sage, Harold P. Erickson

**Affiliations:** 1 Department of Cell Biology, Duke University Medical Center, Durham, North Carolina, United States of America; 2 Department of Biochemistry, Duke University Medical Center, Durham, North Carolina, United States of America; George Mason University, United States of America

## Abstract

**Background:**

*BtubA* and *BtubB* are two tubulin-like genes found in the bacterium *Prosthecobacter*. Our work and a previous crystal structure suggest that BtubB corresponds to α−tubulin and BtubA to β−tubulin. A 1∶1 mixture of the two proteins assembles into tubulin-like protofilaments, which further aggregate into pairs and bundles. The proteins also form a BtubA/B heterodimer, which appears to be a repeating subunit in the protofilament.

**Methodology/Principal Findings:**

We have designed point mutations to disrupt the longitudinal interfaces bonding subunits into protofilaments. The mutants are in two classes, within dimers and between dimers. We have characterized one mutant of each class for BtubA and BtubB. When mixed 1∶1 with a wild type partner, none of the mutants were capable of assembly. An excess of between-dimer mutants could depolymerize preformed wild type polymers, while within-dimer mutants had no activity.

**Conclusions:**

An essential first step in assembly of BtubA + BtubB is formation of a heterodimer. An excess of between-dimer mutants depolymerize wild type BtubA/B by sequestering the partner wild type subunit into inactive dimers. Within-dimer mutants cannot form dimers and have no activity.

## Introduction

Almost all bacteria and archaea have a tubulin homolog FtsZ, which is the major cytoskeletal protein in cytokinesis. Bacterial genes closer to eukaryotic tubulins have been found in *Prosthecobacter* and a few closely related species [1], [2]. They were named BtubA and Btub B, and showed a closer similarity to α and β tubulin (∼35% sequence identity) than to other tubulins or FtsZ [1]. *Prosthecobacter* species, which also possess *FtsZ* [2], probably acquired the tubulin genes by a horizontal gene transfer [1], [3], [4]; their function in the host bacteria is currently unknown.

**Figure 1:**
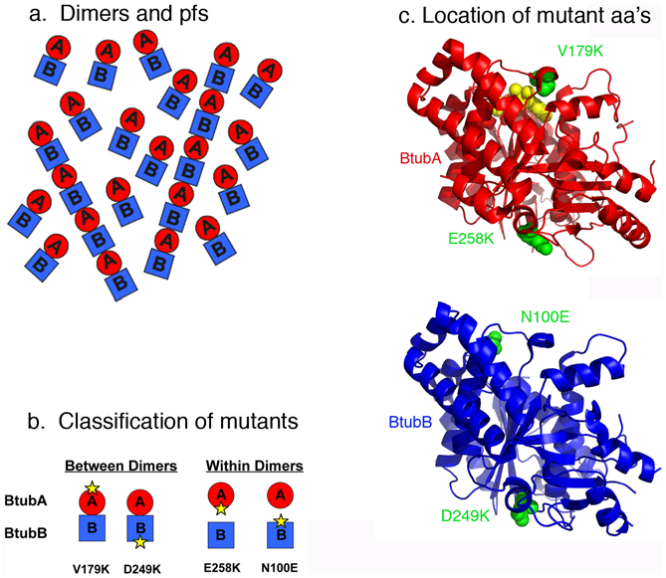
Assembly pathway and location of point mutations in BtubA/B. (a) The proposed assembly pathway in which BtubA and B first assemble into heterodimers, and the heterodimers then further assemble into protofilaments. (b) Two classes of protofilament interface mutants are indicated. One disrupts the interface between dimers, and the other within dimers. The four mutants tested experimentally are indicated. (c) BtubA and BtubB are shown separately in ribbon diagram, and the mutated amino acids are shown in green spacefill. GDP in BtubA is in yellow spacefill. The figures were created in PyMol (DeLano Scientific) from the PDB file 2BTQ [4].

In a previous study [3] we expressed the BtubA and BtubB proteins and showed that they assembled into protofilaments as a 1∶1 mixture. The protofilaments did not form microtubules but instead associated into pairs and bundles that were a few dozen protofilaments thick. Schlieper et al [4] reported similar polymers, and obtained an x-ray crystal structure showing a BtubA/B heterodimer. Although these authors were reluctant to identify which Btub was equivalent to α and β tubulin, we suggest that BtubA corresponds to β tubulin, and BtubB to α tubulin. One justification is that the T7/synergy loop of BtubA closely matches the sequence of β tubulin, including the residue E254, which is K in a tubulin. This loop in BtubB is quite aberrant. Also, the heterodimer in the crystal structure has BtubA at the plus end, the position of β tubulin in the tubulin dimer. Extending this interpretation, the protofilament is thought to form by stacking dimers longitudinally, producing a filament with alternating BtubA and BtubB, with BtubA at the plus end of the protofilament (Fig. 1a).

BtubA/B offers an important advantage for biochemical studies, relative to eukaryotic tubulins, because the proteins can be easily expressed in *Escherichia coli*, and site directed mutants of these proteins can be prepared for in vitro studies. We decided to undertake a mutational approach to characterize the subunit interfaces that form the protofilament. There are two distinctly different interfaces – the one within the dimer, and the one between dimers (Fig. 1b). To study the functions of these interfaces we designed mutations that would disrupt them and determined how the mutations affected assembly and GTP hydrolysis.

## Results and Discussion

### Differential assembly activity of within-dimer and between-dimer mutants

We used the crystal structure of the BtubA/B heterodimer [4], and a previous analysis of subunit contacts in the tubulin protofilament [5], to identify surface amino acids that appeared to be important for longitudinal contacts in the BtubA/B protofilament. We then mutated these amino acids, changing the charge as well as the size of the original amino acid, with the goal of disrupting the interface. We discovered four mutants that were unable to form protofilaments, one each on the top and bottom interface of BtubA and BtubB (Fig. 1b). The locations of these mutants on the crystal structure are shown in Fig. 1c. Two of these mutants are at the interface within the BtubA/B heterodimer, and two are at the interface between heterodimers.

**Figure 2:**
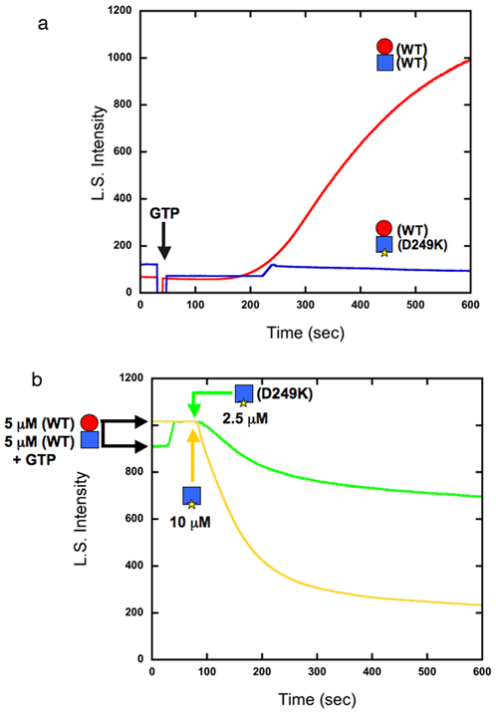
Between-dimer mutant BtubB-D249K fails to assemble and disassembles wild type polymers. (a) Assembly of 5 µM each wild type BtubA plus BtubB is shown by the red curve. The time of GTP addition is indicated. The blue curve shows that wild type BtubA plus BtubB-D249K gave no assembly. (b) polymers were first assembled to steady state from 5 µM each wild type BtubA and BtubB. At the arrows BtubB-D249K was added to 2.5 µM or 10 µM, causing disassembly.

We used light scattering to assay for assembly. The first experiment examined the between-dimer mutant on the bottom of BtubB (Fig. 2). Fig. 2a (red curve) shows assembly of a 1∶1 mixture of wild type BtubA and BtubB. This particular experiment showed a pronounced lag and relatively slow assembly following addition of GTP. In all later experiments the lag is much shorter and assembly more rapid. In this first experiment the GTP was added immediately after mixing BtubA and BtubB, while for later experiments the mixed subunits were allowed to incubate for two min before adding GTP. The pronounced lag shown in Fig. 2 may be due to time needed to form heterodimers. We kept this result for presentation to illustrate the complex kinetics, which we have not yet analyzed in detail. A mixture of wild type BtubB plus BtubA-D249K gave no assembly (Fig. 2a, blue curve).

We then tested whether the mutant BtubA-D249K could disrupt pre-formed wild type protofilaments. We first assembled a mixture of 5 µM each wild type BtubA and BtubB, and when it reached a plateau of light scattering we added BtubA-D249K. Fig. 2b shows that 2.5 µM BtubB-D249K caused partial disassembly of 5 µM wild type polymers, and 10 µM mutant BtubB caused much more extensive disassembly.

**Figure 3:**
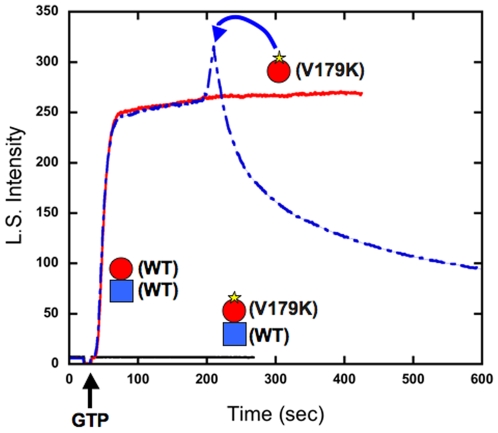
Between-dimer mutant BtubA-V179K. The red line shows assembly of wild type BtubA/B (5 µM each). The black line shows that BtubA-V179K plus wild type BtubB does not assemble. The blue line shows that addition of 5 µM BtubA-V179K to the 5 µM preformed BtubA/B protofilaments caused their disassembly.

The other between-dimer mutant, V179K on top of BtubA, gave very similar results. When mixed 1∶1 with wild type BtubB it gave no assembly at all (Fig. 3). When BtubA-V179K was added to pre-formed wild type polymers, it caused their disassembly. Thus each of the between-dimer mutants caused disassembly when added to wild type polymers.

**Figure 4:**
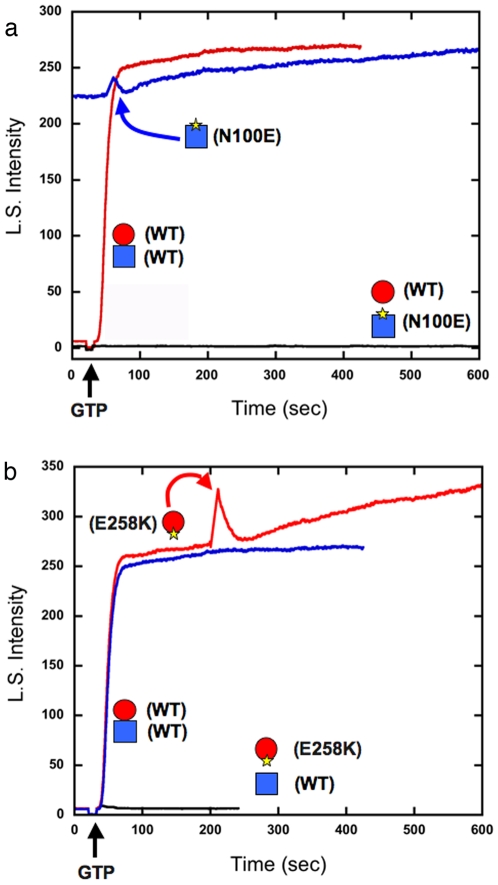
Within-dimer mutants fail to assemble but have no effect on pre-assembled wild type polymers. (a) Within-dimer mutant BtubB-N100E did not assemble when mixed with wild type BtubA (black line). When added to pre-assembled wild type polymer (all subunits at 5 µM) it did not cause any disassembly (blue line). (b) Within-dimer mutant BtubA-E258K behaved the same as BtubB-N100 in both assays.

We then repeated these experiments with mutants located at the interface within the heterodimer. Each of the within-dimer mutants failed to assemble when mixed with a wild type partner (Fig. 4a,b black curves). This is similar to the between-dimer mutants. However, in contrast to the between-dimer mutants, each of these within-dimer mutants completely failed to disassemble pre-formed wild type polymers. E258K showed a slow increase in light scattering, which we have not investigated.

### Effect of the mutations on GTP hydrolysis

Assembly of BtubAB requires GTP, and the GTP is hydrolyzed by the polymers [3], [4]. We assayed each of the mutants, paired with a wild type or mutant partner, for GTPase activity. We first found that the mixture of wild type BtubA plus BtubB gave a much lower rate of hydrolysis than we reported in our previous study, 0.117 GTP min^−1^ Btub^−1^, vs 1.37 previously. We have obtained this lower value in several repeated assays, and we conclude that our previous value may have been affected by contaminating GTPases. We note also that Schlieper et al [4] found that 10 µM BtubA/B assembled rapidly in 500 µM GTP and then disassembled after about 8,000 s as the GTP was hydrolyzed. This suggests a hydrolysis rate of 0.375 GTP min^−1^ Btub^−1^, closer to our present low value than our previously reported rate. Individual BtubA and BtubB subunits gave rates of 0.01–0.02 GTP min^−1^ Btub^−1^, which are near the limit of detection and presumably negligible.

We used two assays to measure GTP hydrolysis. We generally prefer the coupled-regeneration assay [6] for its simplicity and reproducibility, but turbidity produced by polymers interfered with measurements of wild type BtubA/B. For this we used the malachite green assay [7]. The two assays gave similar results for wild type BtubA and BtubB separately (Table 1).

**Table 1:** GTPase activity

**Wild type BtubA/B**	**GTPase** (GTP min^−1^ Btub^−1^)
BtubA + BtubB	.117 mal
BtubA	.017 rgn
BtubA	.014 mal
BtubB	.004 rgn
BtubB	.013 mal
**Mutations between dimers**	
A-V179K + B-wt	.072 rgn
A-wt + B-D249K	.077 rgn
A-V179K + B-D249K	.079 rgn
**Mutations within dimers**	
A-E258K + B-wt	.088 rgn
A-E258K + B-N100E	.098 rgn
	**Hydrolysis of GMPCPP**
A-wt + B-wt	.003 mal
A-V179K + B-D249K	.002 mal
*E. coli* FtsZ	.080 mal

GTPase activities marked rgn were assayed by the GTP regeneration, coupled NADH assay. Those marked mal were assayed with malachite green. The malachite green reactions were measured at room temperature (23°C); the regeneration reactions were at ∼29°C, the temperature in the spectrophotometer chamber). Rates are given per Btub for single subunits, and per BtubB for the 1∶1 mixtures.

The Btub mutants mostly had a GTPase of 0.07–0.09 GTP min^−1^ Btub^−1^ when paired with a wild type or mutant partner. This is less than the pair of wild type BtubA/B, but above the negligible rate of single subunits. This suggests that GTP hydrolysis is stimulated by interaction of monomers capable of forming either within- or between-dimer interfaces, but is increased further when protofilaments can be formed. It is surprising that mutants capable only of forming dimers had GTPase activity, because the synergy loop of the BtubB is so aberrant.

GMPCPP is a non- or slowly hydrolysable GTP analog that supports assembly of tubulin and FtsZ. A light scattering assay (not shown) indicated that it also supported assembly of BtubA/B. We attempted to measure its hydrolysis rate but it was too low to be significant in our assay (Table 1). As a control we measured the rate of hydrolysis of GMPCPP by FtsZ. The value obtained, 0.08 GMPCPP min^−1^ FtsZ^−1^, is 50–70 times slower than hydrolysis of GTP at room temperature. This is substantially slower than the 3–10 X rate reduction previously reported [8]. The previous measurement was made in a buffer at pH 6.5, no potassium, and was noisy and near the limit of detection. The present measurement was made in the more physiological buffer at pH 7.7, 350 mM KAc, and the data are much more reliable.

### Dimer formation assayed by sedimentation equilibrium

We studied the oligomerization of BtubA/B by sedimentation equilibrium, as described in Methods. These experiments were initially done in the absence of GTP, which prevents assembly of protofilaments but may permit assembly of the dimer. Sedimentation of BtubA or BtubB alone gave monomers of the expected molecular weight, with no evidence of dimerization. The data from multiple runs using different ratios of BtubA and BtubB were fit reasonably well by a model assuming an equilibrium between monomer and dimer, and the global fit was used to deduce a K_D_ for dimerization. As shown in Table 2 the wild type protein and the mixture of between-dimer mutants (BtubA-V179K + BtubB-D249K) gave similar K_D_'s of 7.3 and 3.2 µM. The mixture of within-dimer mutants (A E258K + B N100E) gave a K_D_ of 61 µM, suggesting that dimerization is eliminated by blocking the within-dimer interface.

**Table 2:** Sedimentation equilibrium

BtubA	BtubB	K_D_ (dimerization)
wt	wt	7.3 µM
V179K	D249K	3.2 µM
E258K	N100E	61 µM

The sedimentation equilibrium curves were fit to a model assuming an equilibrium of monomer and dimer. The best fit K_D_ for dimerization is shown.

We next used our between-dimer mutants to test whether GTP would affect dimer formation. Instead of GTP we used the slowly hydrolysable analog GMPCPP. The rate of hydrolysis of GMPCPP (0.002–0.003 per min per BtubB, Table 1) would give less than 0.5 µM GMPCPP hydrolyzed by 8 µM BtubA/B in 24 hours. For this experiment we used a simpler data analysis, which treated the mixture as a homogeneous species and estimated a single molecular weight. For a mixture of 8 µM each BtubA-V179K + BtubB-D249K, with no added nucleotide, the estimated average molecular weight was 84 kDa, vs 102 kDa expected for the dimer (protein plus his-tag). This suggests that most of the protein exists as a dimer, as expected for the K_D_ of 3.2 µM. When GMPCPP was added to 16, 32 and 64 µM the estimated molecular weights were 87, 91 and 87 kDa. We conclude that GMPCPP has only a minimal effect on the formation of the dimer.

Schlieper et al [4] also used analytical ultracentrifugation to examine the oligomerization of a mixture of BtubA/B. They concluded that dimers were formed, but their dimerization appears to be weaker than what we found. The difference may be due to the different buffers used in the two studies (e.g., the buffer of Schlieper et al had no Mg, whereas ours had 5 mM Mg). We have not attempted to dissect how buffer conditions might affect dimerization, but note that we used the same buffer for sedimentation, GTPase and all assembly experiments.

The 3-7 µM K_D_ for dimer formation is almost an order of magnitude weaker than the 0.5 µM critical concentration. The mechanism of cooperative assembly that produced the critical concentration is not known, and it is not at all clear how the dimer might fit into the mechanism for cooperativity.

### A search for lateral mutations

In addition to the analysis of mutations that would block the longitudinal protofilament interfaces, we tested a number of mutations on the sides of the subunits, hoping to find ones that might block the association of protofilaments into bundles. The following mutations were made and proteins were successfully expressed and purified: BtubA: S61R, P41R, D283H, E289K and BtubB: E38K, D57H, N217K, R291G. None of these mutations affected assembly of protofilaments or their bundling, as assayed by electron microscopy. In addition we identified the sequence 283DRSKFEELG291 (*P. dejongeii*, AY186779) in BtubA, as corresponding to the M loop that mediates tubulin's lateral bonds [5], and replaced it with the corresponding M loop from β tubulin (*Sus scrofa*, NM001113696) 277GSQQYRALT285. This also had no effect on assembly or bundle formation.

### How between-dimer interface mutants disassemble wild type protofilaments

Both within- and between-dimer mutants were unable to assemble, as expected for mutations that disable a protofilament interface. However, they had very different effects on disassembly of pre-assembled wild type polymers. Between-dimer mutants caused them to disassemble, while within-dimer mutants had no effect. This can be explained by the hypothesis that subunits must assemble into dimers before they can assemble into protofilaments.

**Figure 5:**
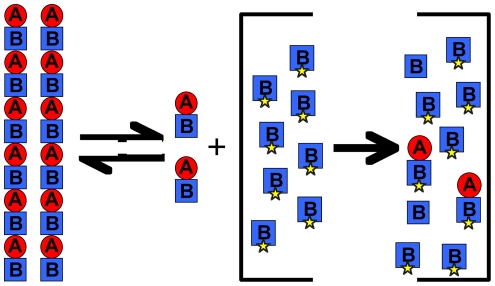
Between-dimer mutants disassemble wild type polymers by sequestering subunits into inactive heterodimers. The equilibrium on the left shows wild type BtubA/B protofilaments exchanging with dimers. These dimers are also exchanging with a small pool of monomers, not shown. The bracket on the right shows what happens when the reaction is flooded with between-dimer BtubB subunits. These are capable of forming dimers, and because they are in excess of the monomer/dimer pool they will replace the wild type BtubB. This sequesters most of the wild type BtubA into inactive dimers. This depletion of wild type dimers to below the critical concentration results in depolymerization.

In an assembly mixture of wild type BtubA/B, polymers will be in steady state exchange with dimers. The dimers will be in a separate equilibrium with monomers. In this monomer-dimer pool, BtubA and BtubB will each be maintained at the critical concentration of ∼0.5 µM (as determined in [3]) (Fig. 5). When an excess of between-dimer mutant BtubB is added, it will exchange with the wild type BtubB in the monomer/dimer pool and form dimers with the wild type BtubA. Because the mutant BtubB is in large excess over the 0.5 µM pool of monomer/dimer BtubA, most of the BtubA will be sequestered into dimers that cannot assemble. As the pool of active wild type dimers drops below the critical concentration, protofilaments will disassemble. As more dimers are released, the wild type BtubA will continue to be sequestered by the mutant BtubB. The disassembly is driven not by direct interaction of the mutant BtubB with protofilaments, but by its sequestering wild type BtubA into inactive mutant dimers.

The within-dimer BtubB mutant, in contrast, cannot form a dimer with wild type BtubA. It remains as a monomeric subunit that is inactive for assembly and cannot sequester wild type BtubA. It is therefore unable to disassemble preformed wild type polymer (Fig. 5).

### Implications for α−β tubulin

The *btubA* and *btubB* genes were likely acquired by *Prosthecobacter* by horizontal gene transfer of α− and β−tubulin from a eukaryotic host [1], [3], [4]. Both genes would apparently have been transferred at once, which suggests that the host species had α− and β−tubulin in tandem arrays, as occurs in trypanosomes [9]. (However the *BtubA* and *BtubB* genes in the known *Prosthecobacter* species all have a kinesin light chain gene separating them, which is not the case for arrays in eukaryotes.) The proteins would initially retain the ability to assemble microtubules, but since microtubules were presumably not functional in *Prosthecobacter* they would be free to diverge. They apparently retained the longitudinal interfaces that enable assembly of protofilaments, but lost the lateral bonding interfaces that assemble protofilaments into the microtubule wall.

One problem with this scenario is that all known eukaryotic tubulins require complex chaperones for folding [10]. It is unlikely that these chaperones would be transferred with the tubulin genes, so how could the newly acquired tubulins fold in the *Prosthecobacter* cytoplasm? One possibility is that bacterial chaperones might have sufficed for folding. We note, however, that there is no convincing case of a eukaryotic tubulin being expressed and folded in *E. coli*, so *E. coli* chaperones are not sufficient for folding. Another possibility is that the tubulin genes came from a host that did not require chaperones for folding. Again, no such species is known today. Whatever the beginnings, it is clear that no chaperone is necessary for folding the present-day BtubA and BtubB. They fold properly in the foreign host *E. coli*, and they can be refolded in vitro after chemical denaturation [4].

The lateral bonding that assembles tubulin protofilaments into the microtubule wall has been lost in BtubA/B, but the longitudinal bonding that assembles subunits into protofilaments has been preserved. In tubulin these longitudinal contacts are of two types. The bond within the dimer is almost irreversible, with K_D_ near pM, and an exchange half time of hours [11]. The bond between dimers is much weaker, with K_D_ near mM (it must be supported by a lateral bond to produce significant assembly) [12], [13]. An important conclusion from the present work is that BtubA/B has maintained the ability to form heterodimers. The within-dimer bond is orders of magnitude weaker than that of tubulin, but still sufficient to assemble a pool of heterodimers that then assembles into protofilaments and bundles. The nature or geometry of lateral bonds holding the protofilaments in bundles is not yet clear, so the magnitude of between-dimer bonds cannot be estimated. It is presumably weaker than the within-dimer bond, which forms first, and relies on some mechanism of cooperativity to produce assembly. The heterodimer appears to be an essential intermediate in the assembly of BtubA/B, as it is for tubulin.

## Materials and Methods

### Growth and induction

N-terminally his-tagged BtubA and BtubB was expressed in *E. coli* and purified as described previously [3]. For the present study we added another purification step, running the proteins over a Sephacryl HR-100 column equilibrated with HMK buffer (50 mM Hepes, 5 mM MgAc, 350 mM KAc, and 1 mM EGTA, pH 7.7). This buffer was used for all experiments. Peak fractions were stored at −80°C. Protein concentration was determined from the absorbance at 280 nm; the extinction coefficients were 4.790×10^4^ M^−1^ cm^−1^ for BtubA and 3.988×10^4^ M^−1^ cm^−1^ for BtubB, based on amino acid composition [14], [15]. The extinction coefficient of GDP is 0.9×10^4^ at 280 nm, and we previously found that BtubA and B bound 0.7 and 0.3 mol GXP [3]. By ignoring the contribution of GXP we may have underestimated the protein concentration by 7–13%.

Schlieper et al [4] reported that “his-tags are not necessary but interfere with polymerization.” We have tested several preparations with and without his-tags. We did observe that assembly of rings by BtubB alone was substantially reduced by the removal of the his-tag. However, we found no difference in the polymerization of protofilaments and bundles with and without the his-tags. All experiments reported here were done with the his-tags in place.

### Electron microscopy

Negatively stained samples were prepared by applying ∼10 µl of the assembled BtubA/B to a carbon-coated grid and washing off with 3–4 drops of 2% aqueous uranyl acetate. Electron micrographs were taken at 50,000x.

### GTPase Assay

To measure GTPase activity we used the continuous, regenerative coupled GTPase assay of Ingerman and Nunnari [6]. Our assay mixture included 0.4 mM phosphoenolpyruvate, 0.3 mM NADH, 20 U/ml each pyruvate kinase and lactate dehydrogenase (Sigma), and 0.5 mM GTP. Each GDP released from BtubA/B is regenerated to GTP with the loss of one molecule of NADH. NADH concentration was monitored by its absorbance at 340 nm (extinction coefficient 6220 M^−1^ cm^−1^) using a Shimadzu UV-2401PC spectrophotometer. Following addition of GTP, the absorbance showed a linear decrease over time. We measured the slope of the straight line at steady state, typically between 100 and 600 sec after addition of GTP. As in our previous study [3] the GTPase of wild type BtubA/B was only significant above a critical concentration of ∼0.5 µM. A critical concentration was less obvious for the lower GTPse of the mutant proteins. In all cases we measured GTPase over a range of concentrations, and the slope of the straight line (above the 0.5 µM critical concentration for wild type) gave the overall rate of GTP hydrolysis in GTP per min per BtubB. In some cases we used a malachite green assay [7]. Measurements were made in HMK buffer at room temperature (∼23°C) for the malachite green reaction; the regeneration assays were at ∼29°C due to warming in the chamber of the spectrophotometer.

### Assaying polymer by 90 degree light scattering

Varying concentrations of a 1∶1 molar ratio of BtubA and BtubB in HMK buffer (total volume of 100 µL) were loaded in a quartz cuvette with a 1-cm path length. The cuvette was placed in a Shimadzu fluorometer that had both the excitation and emission wavelengths set at 350 nm and at varying slit widths of 3 to 5 nm. A baseline of scattering for the protein mixture without added GTP was established for 20 s and then polymerization was initiated by the addition of 1 mM GTP. The nucleotide was added with a pipette followed by mixing. The elapsed time between nucleotide addition and the start of the recording was typically 5 s. The net change in light scattering after nucleotide addition was recorded until a plateau representing the polymerized BtubAB protofilaments was established. Except as noted BtubA and BtubB were 5 µM in all assembly and disassembly experiments. Measurements were made in HMK buffer at room temperature (∼23°C).

### Sedimentation Equilibrium

Sedimentation equilibrium analysis was performed at 20°C at 10,000, 12,000 and 14,000 rpm using a Beckman Optima XL-A analytical ultracentrifuge equipped with a 60Ti rotor and six channel centerpieces. Protein samples were in HMK buffer. We examined mixtures of wild type and mutant proteins with molar ratios for BtubA∶BtubB ranging from 1∶4 to 4∶1 (the concentration of each protein ranged from 8 to 32 µμM, and were 8 µM each at the 1∶1 ratio). Cells were scanned at 6 hour intervals at 280 nm until consecutive scans (typically three) were unchanged and the system was judged to be at equilibrium (typically 24 h). The data from each set of experiments (BtubA WT + BtubB WT, BtubA-V179K + BtubB-D249K, and BtubA-E258K + BtubB-N100E) were globally fit with the program HeteroAnalysis v 1.1.0.28 of James L. Cole (http://www.biotech.uconn.edu/auf/) to obtain the dissociation constant for dimerization (Table 2). Partial specific volume values of 0.736, 0.733, and 0.734 corresponding to BtubA, BtubB, and mixtures of BtubA and BtubB, were all based on amino acid composition using the program SEDNTERP [16]. The program SEDNTERP was also used to calculate the value of 1.0142 for the density of the solvent. In a separate set of experiments examining the effect of GMPCPP, the sedimentation equilibrium data were analyzed using the Ideal-1 program (Beckman Instruments). This treats the protein as a homogeneous species and gives a single estimated molecular weight.
